# FangTianSim: High-Level Cycle-Accurate Resistive Random-Access Memory-Based Multi-Core Spiking Neural Network Processor Simulator

**DOI:** 10.3389/fnins.2021.806325

**Published:** 2022-01-20

**Authors:** Jinsong Wei, Zhibin Wang, Ye Li, Jikai Lu, Hao Jiang, Junjie An, Yiqi Li, Lili Gao, Xumeng Zhang, Tuo Shi, Qi Liu

**Affiliations:** ^1^Zhejiang Laboratory, Institute of Intelligent Computing, Hangzhou, China; ^2^Institute of Microelectronics, Chinese Academy of Sciences, Beijing, China; ^3^School of Microelectronics, University of Science and Technology of China, Hefei, China; ^4^Frontier Institute of Chip and System, Fudan University, Shanghai, China

**Keywords:** spiking neural network (SNN), RRAM (memristor), simulator, analog circuits, SystemC

## Abstract

Realization of spiking neural network (SNN) hardware with high energy efficiency and high integration may provide a promising solution to data processing challenges in future internet of things (IoT) and artificial intelligence (AI). Recently, design of multi-core reconfigurable SNN chip based on resistive random-access memory (RRAM) is drawing great attention, owing to the unique properties of RRAM, e.g., high integration density, low power consumption, and processing-in-memory (PIM). Therefore, RRAM-based SNN chip may have further improvements in integration and energy efficiency. The design of such a chip will face the following problems: significant delay in pulse transmission due to complex logic control and inter-core communication; high risk of digital, analog, and RRAM hybrid design; and non-ideal characteristics of analog circuit and RRAM. In order to effectively bridge the gap between device, circuit, algorithm, and architecture, this paper proposes a simulation model—FangTianSim, which covers analog neuron circuit, RRAM model and multi-core architecture and its accuracy is at the clock level. This model can be used to verify the functionalities, delay, and power consumption of SNN chip. This information cannot only be used to verify the rationality of the architecture but also guide the chip design. In order to map different network topologies on the chip, SNN representation format, interpreter, and instruction generator are designed. Finally, the function of FangTianSim is verified on liquid state machine (LSM), fully connected neural network (FCNN), and convolutional neural network (CNN).

## Introduction

The success of artificial intelligence technology represented by deep neural network (DNN) today depends heavily on the development of big data and chip technology. However, the problem of DNN lies in its massive parameter volume, leading to high energy consumption. Therefore, people are exploring energy-efficient artificial intelligence algorithms. Inspired by the characteristics of biological brain, such as asynchrony, and being event driven, spiking neural networks (SNNs) are considered to have the potential to realize ultra-low power intelligent computation (Maass, 1997). At present, SNNs can achieve similar results with DNNs in some small-scale applications, based on various training algorithms, e.g., BP (Esser et al., 2015), spike-timing-dependent plasticity (STDP) (Neftci et al., 2014), and network conversion (Diehl et al., 2015). In addition, compared with DNN, SNN is better at processing spatiotemporal information. For example, the performance of SNN in tasks, e.g., dynamic gesture recognition and language feature extraction, is equivalent to DNN with the same structure (Blouw et al., 2019). In order to run SNN efficiently, dedicated chips with asynchronous operation and being event driven have been widely studied. In the early stage of SNN chip research, analog circuit was used to realize a neuron model (Silver et al., 2007) and cooperate with digital communication systems, such as network-on-chip (NoC) to realize the chip (Boahen, 2006; Schemmel et al., 2008; Qiao et al., 2015). Although this chip runs SNN with low power consumption, its function is very limited, and it is mostly used to study a small-scale brain model. In recent years, people use advanced semiconductor technology and advanced asynchronous circuit design technology to realize large-scale integrated SNN chips. These chips not only have high energy efficiency but can also realize complex neuron models and a variety of synaptic plasticity. Good results have been obtained in the fields of handwritten character recognition, dynamic vision sensor (DVS) gesture recognition, and small sample gas classification (Akopyan et al., 2015; Davies et al., 2018; Deng et al., 2020).

However, due to the limitations of complementary metal oxide semiconductor (CMOS) technology and circuit design methods, both the analog–digital hybrid SNN chip and SNN chip based on asynchronous circuit are far from the scale of biological brain. To further increase the energy efficiency and integration of the chip, researchers have focused on emerging RRAM (Strukov et al., 2008). At present, small-scale SNN based on RRAM array has been verified (Fang et al., 2020; Lu et al., 2020; Shi et al., 2021). Owing to its multi-level resistance states, RRAM is also used to realize the SNN chip with *in situ* learning ability (Jo et al., 2010). However, current RRAM-based SNN chips are small-scale and designed for fixed network structures. It is still challenging to realize large-scale reconfigurable chips based on RRAM. The origins are the non-ideal characteristics of RRAM, and the complexity of cross-level design and optimization (device-circuit-architecture-algorithm) restricts the performance of networks. Solving the above problems not only depends on the continuous improvement of the RRAM device and circuit design but also heavily depends on the development of simulation tools for cross-level design and optimization. In order to break the barrier between SNN algorithm, hardware architecture, circuit and RRAM devices, some simulation tools have been developed, such as MNSIM for the behavior level modeling of device and circuit (Xia et al., 2017). The simulator can estimate the area, power consumption, and delay of the RRAM chip according to the actual process, but this work lacks the research on the architecture and algorithm levels. NeuroSim and some works based on it (Chen et al., 2018; Peng et al., 2019; Wu et al., 2019) and Neurosim+ (Chen et al., 2017) provide modeling from device level to circuit and algorithm level. However, the tool directly jumps from circuit level to algorithm level. Although it complements the discussion of algorithm, it lacks the ability to analyze chip architecture. PIMSim (Xu et al., 2018) provides a tool to understand configurable PIM. It supports a variety of PIM models, supports instruction execution, and simulates the PIM system from the system architecture level. However, these works still have two disadvantages. One is the lack of support for SNN algorithm, and the other is the inability to carry out clock cycle level simulation, so it is impossible to explore the impact of pulse transmission delay on the network. For some NPU simulators, the software and hardware collaborative design language SystemC (Panda, 2001) is used to model the circuit architecture, and system architecture simulation at the clock level is realized, such as NN-Noxim (Chen and Wang, 2018), Noxim (Catania et al., 2015), etc. However, SNN and RRAM are not included in these works.

According to the requirements of designing large-scale reconfigurable SNN chip based on RRAM, we provide a simulator FangTianSim that can conduct behavior level modeling for RRAM-based SNN architecture. The simulator can conduct behavior level modeling for NoC, spiking neurons, and RRAM arrays. Operation speed, delay, and function can be simulated in the digital domain with accuracy to the clock cycle level. This tool can save a lot of time and simulation resources and guide chip design effectively. The contributions of this paper mainly include a clock cycle level simulator for RRAM-based SNN chip architecture, power consumption analysis method based on actual process, and network parsers and instruction generation tools for a variety of SNN architectures.

## Spiking Neural Network and Hardware Architecture

### Spiking Neural Network

In SNN, spiking neurons with integration and fire characteristics are used as nodes in the network, and synapses with weight are used to link neurons. The pulse signal generated by neurons is the main medium of network information transmission. At present, the mainstream neuron models include Hodgkin-Huxley (HH) (Abbott, 1999), leaky integrate-and-fire (LIF) (Hodgkin and Huxley, 1952), Izhikevich (IZH) (Izhikevich, 2003) model, etc. Considering the computational complexity and accuracy of neurons, most neurons in SNN adopt the LIF model. The LIF model is shown in the following formula:


$$\frac{d\,V}{d\,t}=\gamma \,V+u$$

$$s\,p\,i\,k\,e=\left\{ \begin{array}{c} 1\,V>V_{t\,h}\\ 0\,V\leq V_{t\,h} \end{array}\right.$$

where *V* denotes membrane potential, t denotes time, γ denotes the leakage rate, *u* denotes input and *V*_*th*_ denotes fire voltage. Due to the use of sparse asynchronous pulses for information processing, SNN has three advantages: (1) the bandwidth and energy consumption required to transmit binary pulses are far less than the continuous value of multiple bits; (2) when calculating a single bit input, neurons only need to add the synaptic weight to the membrane potential, while DNNs need to multiply and accumulate the input and weight; (3) SNN adopts an event-driven approach. When there is no input pulse, the neurons are in a resting state, while the neurons in DNN need to be in a working state all the time.

### Hardware Architecture

**FIGURE 1:**
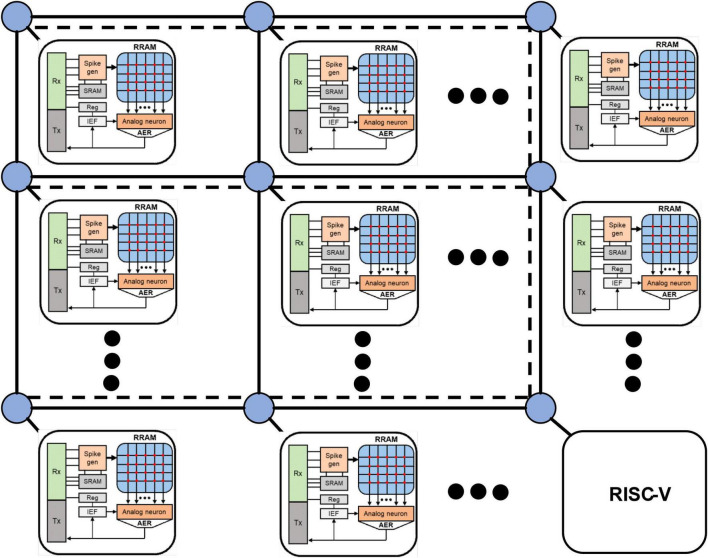
SNN chip architecture. The SNN cores in the simulator consists of analog neurons, RRAM dendrites, SRAM axons and digital control circuit. The output of the neuron is converted into an address signal by an address event representation (AER) circuit. The entire SNN chip structure consists of 63 such SNN cores and a RISC-V instruction set microcontroller.

The main body of SNN chip is usually composed of spiking neuron circuit, synaptic memory, and flexible on-chip communication system. In order to make full use of the advantages of SNN, the NoC system is often used to realize the interconnection between neurons. The introduction of RRAM synapse and analog neuron circuit into the design of the SNN chip can further improve integration and energy efficiency. The SNN chip architecture simulated by FangTianSim consists of 63 SNN cores and a microcontroller. Each core is composed of 32 analog neurons, 8 K RRAM dendrites and a set of SRAM axons, as well as digital control circuits. The simulated neurons adopt the LIF model to support positive and negative weights; the output of 32 neurons is converted into an address signal through an address event representation (AER) circuit. The SNN chip adopts mesh NoC. Mesh NoC is compatible with clock-gate and is relatively simple, leading to low area and power consumption. Therefore, it is very suitable for SNN chips. The chip architecture is shown in Figure 1. The neuron circuit adopts the analog LIF neuron circuit. The RRAM array with 2T1R structure is used to store synaptic weights. The mirror current source circuit is used to make the neuron support the output current of RRAM cells representing positive and negative weights. The common mode feedback circuit of the differential amplifier is used to make the neuron support the bidirectional leakage circuit to ensure the leakage mode and make the membrane potential tend to the resting potential.

In addition to digital circuits, there are analog neuron circuits and RRAM synapses in the chip. The scale and design complexity of the chip are very high. Therefore, FangTianSim is developed to save resources and time in chip design. The simulator simulates the inference process of the network; in this process, the influence of read noise is added as non-idealities to better simulate the real situation in hardware. According to the non-idealities of the RRAM used, the noise in the simulator is mainly divided into two parts, the first part is the noise which includes thermal noise (Gaussian distribution), shot noise (Poisson distribution), and RTN noise (Poisson distribution) added on the RRAM arrays; the second part is the noise added on the neuron threshold (Gaussian distribution).

## Simulator Design

### Difficulties and Solutions

In the SNN algorithm running in the software, the time when the pulse generated by the neuron of each layer reaches the neuron of the next layer is synchronous, i.e., the pulse reaches the next layer immediately after the pulse is generated (or arrives at the next unit simulation time). However, in the chip, the pulse generated by the first layer needs to be transmitted to the next layer through a router. As a result, the delay of pulse transmission between different cores is different, which is related to the Hamming distance between the two cores. In addition, because the pulse generated by multi-channel neurons needs to be converted into serial signal through the AER circuit, a pulse transmission delay will also be generated in this step. When the router is congested, the pulse transmission delay is more difficult to estimate; therefore, the SNN algorithm run by hardware will be different from that run by software. Analog neurons have a time constant controlled by voltage or current, which will control the speed of leakage, and this constant cannot be accurately quantified in the circuit (accurate quantization or control of time constant requires more complex circuits, which will consume a lot of area and power consumption, so it is inconvenient to use in analog neurons). Since the SNN chip based on analog neurons does not control the synchronization of pulses, whether the propagation delay of pulses affects the results of the neural network must be considered when designing the chip and the analysis of this impact will be particularly important. Due to the high complexity of the SNN chip, it is necessary to design a simulator for chip design, and consume less resources and running time to verify the function and performance of the whole chip. In particular, the simulation speed must be fast enough to simulate the actual use case of the SNN algorithm in a limited time, and the simulation must accurately reflect the pulse delay caused by the actual chip operation. In order to implement the simulator described above, this section proposes a chip system simulator FangTianSim based on C++ and SystemC. In order to cooperate with the operation of the simulator, a simple tool chain is designed for the chip.

### Simulator Architecture

**FIGURE 2:**
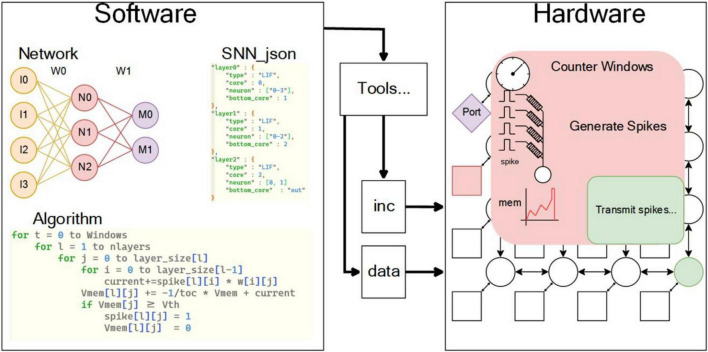
Software and hardware for SNN. The software part mainly includes the description and algorithm of neural network, and the hardware part mainly includes NoC-based interconnection and neuron dynamic change, which interact with each other through specific tool chain.

Rational division of software and hardware functions is an important work in designing complex chips. When designing the SNN chip, the first thing is to analyze the SNN architecture: As shown in Figure 2, the structure of neural network is hierarchical, and neurons from adjacent layers are interconnected by synapses. According to the algorithm workflow code, the SNN algorithm runs according to cycle of simulation time in software operation. Each cycle represents a unit time of neural network operation (called unit time window), and the subsequent cycles are network level, pre neuron and post synaptic neuron, and the input current and simulation unit of each neuron are calculated in turn. When the hardware runs the network, the counter is used to control the time length of the unit time window. The levels of the network are assigned to different cores, and the interconnection between cores is used to represent the interconnection of different levels. Current calculation and membrane potential calculation are handed over to memristor array and analog neuron, respectively. According to the above discussion, the network task is divided into two parts. Software part: (1) network construction; (2) network training—the network used in the simulation was trained with back propagation algorithms (BP) (Esser et al., 2015; Zhang et al., 2015); (3) trained network structure and weight according to SNN_ JSON format storage; (4) using the specific tool of chip to convert the network structure; (5) weight into hardware recognizable instructions; and (6) the socket of the on-chip network, and the input pulse is also converted into pulse socket through the tool chain. Hardware part: (1) network hardware connection (NoC-based interconnection), (2) input current calculation (in memory calculation based on RRAM), and (3) neuron dynamic change (neuron membrane potential, action potential, refractory period processing).

**FIGURE 3:**
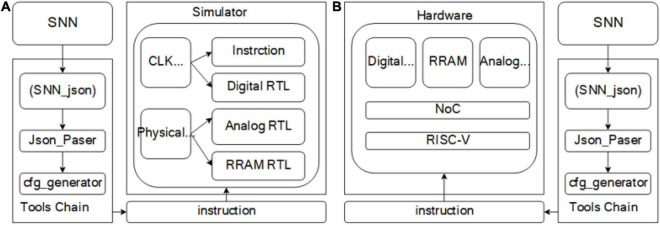
**(A)** Software system. **(B)** Hardware system. The software system and hardware system share a set of tool chain, which is composed of SNN_JSON, JSON interpreter and instruction generator. After the tool chain generates a machine code, it is transmitted to the system through the instruction delivery module.

According to the above division, the complete tool chain and simulation platform are shown in Figure 3. The tool chain is controlled by SNN_JSON, JSON interpreter, and instruction generator; SNN_JSON and SNN structures that meet the hardware constraints of this chapter can be represented by JSON in the tool chain. The interpreter constructs a graph model that can be recognized by the tool chain, which is described by C++ classes, and the instruction generator generates the machine code that can be recognized by the hardware. The machine code is transmitted to the simulator through the instruction distribution module. Developed according to SystemC, the simulator accurately simulates the timing, logic, and delay of hardware through a clock-based time trigger mechanism (CLK EDI). In addition, the model of analog circuit and RRAM circuit is designed with self-defined physical time trigger mechanism (physical EDI). The physical EDI used in this section is a higher-speed CLK EDI. The RRAM model adopts the statistical model actually tested in our laboratory (Lu et al., 2020) and the basic resistance plus random noise. The model of analog circuit is the behavior model of analog circuit, and the circuit voltage change rate of the critical path is obtained with simulation. The PDK of the module is SMIC180, and the *R*_*on*_/*R*_*off*_ of RRAM is 10 KΩ/100 KΩ; read voltage and read pulse-width of RRAM are configurable and the defaults are 0.1 V and 200 cycles (50 MHz). The parameters of SRAM are obtained by querying in SMIC IP according to PDK; the default of PDK is SMIC180. By default, the power consumption of SRAM read/write operations is 7.38 and 9.54 μJ, respectively, and the power consumption of pulse generation is 0.0078 μJ. The above model can accurately simulate the hardware running speed, delay, and function in the digital domain, in which the delay and speed can be accurate to each cycle; However, the model in the simulation domain can only simulate the function and part of the noise. As shown in Figures 3A,B, the tool chain for the simulator (software system) can be directly used for subsequent chip testing (hardware system). In addition to the simulation of function and timing, power analysis is also added to the simulator, which can analyze the read–write power consumption of SRAM, read–write power consumption of RRAM, digital domain and analog power consumption when the neural network is running. The simulator evaluates the power consumption of each part by counting the number of different operations in the network inference process. These include SRAM write/read operations, operations in which neurons accumulate charges and generate a pulse, and operations in which current flows through cells in RRAM. The power consumption of SRAM write/read operation is obtained by querying the SMIC IP, the power consumption of pulse generation is obtained by neuron circuit simulation, and the power consumption of RRAM is obtained by the calculation of current and conductance in specific cells within the time slice. The power consumption analysis of digital domain and analog domain is calculated by the average power consumption provided by traditional EDA tools. The power consumption of SRAM and RRAM can be accurately simulated according to the operation times of SRAM and RRAM by the simulator. The main input parameters of the simulator include network structure, mapping structure, weight value, number and size of RRAM arrays, specifications of RRAM and SRAM and on-chip buffer size. The outputs of the simulator include timing, data flow, and energy of each module and the entire system accurate to each cycle.

## Mapping Neural Network to Chip

### Mapping Method

**FIGURE 4:**
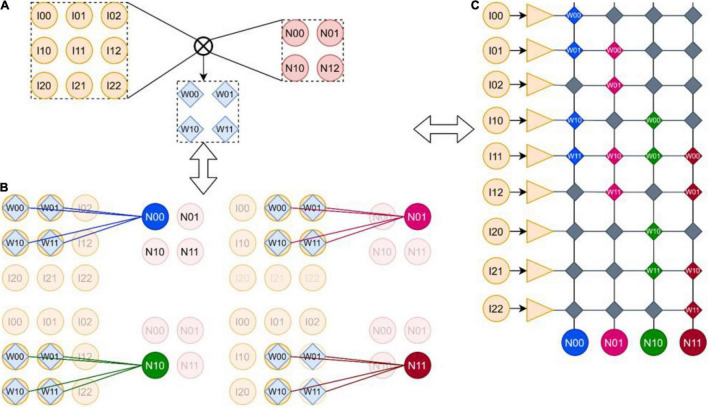
**(A)** Convolution kernels. **(B)** Convolution process. **(C)** The mapping of convolution in memristor array. One 2 × 2 convolution kernel strides successively on the 3 × 3 input feature image, and outputs a 2 × 2 feature image; the convolution layer can be mapped to RRAM but wastes a lot of area.

There are usually three kinds of connection relations of SNN: FCNN, CNN, and recurrent neural network (RNN). The connection mode of SNN is similar to that of DNN, so the representation format for SNN can be designed with reference to the above format. Therefore, FangTianSim can be used to analyze the operation of typical SNN structures in the hardware architecture. In this chapter CNN is taken as an example (Figure 4). The mapping of other SNN structures can be found in Supplementary Materials, where Supplementary Figure 1 corresponds to two-layer FCNN structure and Supplementary Figure 2 corresponds to RNN structure.

In FangTianSim, all these mapping structures are described by the SNN_JSON file: it includes two major classes: Reglist and Layer. Reglist stores the parameters of each core, including SW, PW, ref, reft, window, step, debug, and other members; Layer is used to define the hierarchy of neural network. This class can be defined based on the neural network structure on algorithm or hardware. The layer includes type, core, neuron and bottom_core and bottom_synapse, and other information. Type refers to the type of nerve and can be “LIF” or “IF”; core refers to an independent layer in the corresponding hardware core or algorithm. Neuron defines the number of neurons, which can be a single number to represent the number of neurons. A list is specific to the label of each neuron, and bottom_core refers to the label of the next layer neural network or the next hardware core pointed to by this layer; bottom_ synapses refers to the label of dendrites in the next hardware core, which can be a number or a list. An SNN_JSON example is shown in Table 1.

**TABLE 1:** Configuration list of SNN_JSON.

Class	Parameter	Value	Comment
RegList	Sw	4	Low level time of input pulse
	Pw	14	High level time of input pulse
	REF	1	Types of refractory periods
	REFT	0	The length of the refractory period
	timer_window	200	Time window size
	timer_step	2	Time steps
	output_core	0	The label of output core
Layer1	Type	LIF	Type of neuron
	Core	0	An independent layer in the corresponding hardware core
	Neuron	0–31	Number of neurons
	bottom_core	1	The label of the next layer
	bottom_synapse	0–31	The label of dendrites in the next hardware core
Layer0	Type	LIF	Type of neuron
	Core	1	An independent layer in the corresponding hardware core
	Neuron	0–31	Number of neurons
	bottom_core	4	The label of the next layer
	bottom_synapse	32–63	The label of dendrites in the next hardware core

### Convolutional Neural Network

Figure 4 shows the mapping of CNN on the SNN chip. A 2 × 2 convolution kernel (Figure 4A) is translated successively in the 3 × 3 input feature image. Each translation multiplies and adds the convolution and covered feature image with the weight of the convolution kernel and finally outputs a 2 × 2 feature image (Figure 4B). The mapping of CNN in the memristor array is shown in Figure 4C; it consists of flattening the input image into a one-dimensional input array and flattening the neurons with output characteristics into a one-dimensional output neuron array. Then, a column of synapses connected to each neuron is corresponding to the calculation of convolution operation, such as the N00 neuron; the synapse corresponding to I00 is configured as W00, the synapse corresponding to I01 is configured as W01, the synapse corresponding to I10 is configured as W10, the synapse corresponding to I11 is configured as W11, and other synapses are configured as 0. Other neurons, by analogy, have similar configuration methods. As can be seen, the above configuration method will waste a lot of RRAM, but due to the synaptic array of the crossbar structure and the adoption of analog neurons (the analog neuron circuit needs to connect the analog neurons directly with the synaptic array, and the configurable analog interconnection method needs to occupy a very high area and power consumption), this configuration method is difficult to improve, However, due to the high density of memristors, 3D memristor arrays can even be integrated in the future. These defects can be made up by using the synaptic array of memristors. In addition, through the optimization algorithm, we can try to design a CNN suitable for crossbar structure and also optimize the defects caused by these mapping methods. A CNN suitable for crossbar will be introduced in the later chapter to recognize handwritten characters.

## Results

In this summary, LSM, FCNN, and CNN are verified in the simulator and the simulation results are shown in Table 2.

**TABLE 2:** Simulation results of FangTianSim.

Network	Data set	Latency	Total power consumption	Power consumption (pulse)	Power consumption (RRAM)	Power consumption (SRAM)	Recognition accuracy
LSM	FSDD	5.6 ms	5.74 mJ	34.4 μJ	1.4 μJ	5.7 mJ	83%
FCNN	MNIST	48.5 μs	9.02 μJ	0.249 μJ	0.001 μJ	8.77 μJ	82%
CNN	MNIST	76 μs	12.45 μJ	0.95 μJ	1.4 μJ	10.01 μJ	95%

### Liquid State Machine

The structure of LSM (Zhang et al., 2015) network is composed of 512 neurons and arranged into a 16 × 4 × 8 (*z* = 16, *x* = 4, *y* = 8) cube. The neurons of each layer in the z-axis direction of the cube correspond to a core. Since the LSM network is used to recognize digital speech, the data set adopts free spoke digital dataset (FSDD). As we know from the above, the recognition of sound signals requires operations such as filtering and coding, so it is written in MATLAB with a variety of built-in wave recorders, and generates an SNN_ JSON file. The experimental results show that the code written by MATLAB needs 104 ms to recognize a sound signal (filtering and coding are not included). The simulation results of the simulator show that if it runs under our simulator, it takes 5.6 ms and consumes 5.74 mJ energy, including 34.4 μJ for pulse, 1.4 μJ for RRAM, and 5.7 mJ for SRAM.

### Two-Layer Fully Connected Neural Network

The two-layer FCNN structure: the input layer of the first layer is divided into four blocks, which are input into four different cores and form an intermediate layer. The neurons in the middle layer are input into 10 neurons in the output layer. The input to the intermediate layer and the output layer are also offset pulses (and bias); the schematic is in Supplementary Figure 3. The final simulation results show that the SNN written by Pytorch runs a use case for 400 μs, and the recognition rate is 95.25%. The program runs using Tesla V100 GPU launched by NVIDIA. The simulation results of FangTianSim show that the time for the hardware to run a use case is 48.5 μs, and the generated energy consumption is 9.02 μJ, of which the pulse energy consumption is 0.249 μJ, the RRAM energy consumption is 0.001 μJ, and the SRAM energy consumption is 8.77 μJ. Due to the excessive number of MNIST data sets, the recognition rate of the whole data set is not verified in FangTianSim, but for the 20 randomly sampled use cases, The recognition rate of FangTianSim running the network model is similar to that of Pytorch running the network model.

### Convolutional Neural Network

**FIGURE 5:**
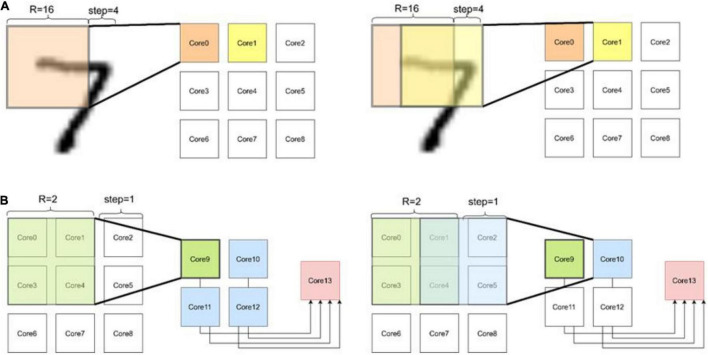
CNN for MNIST. This is the operation process of a three-layer network on the chip. **(A)** In the first layer, the convolution results generated by each slide are put into a separate core. **(B)** The second layer convolved the data of four adjacent cores simultaneously. Finally, all the generated data is routed to one core for full connection process.

As shown in Figure 5, the neural network based on convolution has a three-layer neural network (Esser et al., 2015), and the first layer adopts kernel = 16 step = 12; the second layer adopts kernel = 2, step = 1; the third layer is 10 output neurons. The algorithm is implemented by Pytorch and run by Tesla V100 GPU. The operation results show that the recognition rate at the retest level can reach more than 96%, and the average time to run a case is 126 μs. The simulation results of FangTianSim show that the time for the hardware to run a use case is 76 μs, and the generated energy consumption is 12.45 μJ, including 0.95 μJ for pulse, 1.4 μJ for RRAM, and 10.01 μJ for SRAM.

### Performance

According to the measured results, it takes about 2 min and 50 s for FangTianSim to run the two-layer FCNN designed in this section, while it takes about 15 min and 47 s for the verilog compiled simulator (VCS) software provided by Synopsys to simulate the chip (also running the two-layer FCNN). As shown in Table 2, it can be concluded that the running time of FangTianSim is shorter than that of accurate simulation through VCS. Therefore, using FangTianSim proposed in this work for chip architecture verification can effectively save simulation time, but accurate logic circuit simulation and analog circuit simulation still need to rely on professional simulation tools, such as VCS and SPICE.

## Summary

FangTianSim is a specific simulator based on specific architecture written by SystemC. It can realize clock-level simulation through event trigger mechanism. Through FangTianSim, one can quickly and accurately obtain the operation of different neural networks in the hardware architecture and use it to analyze and optimize the hardware architecture. FangTianSim can count the number of pulses, routing load, SRAM read–write frequency and RRAM read–write frequency. Moreover, because FangTianSim has clock-level simulation accuracy, the impact of pulse transmission delay on SNN can be estimated through FangTianSim. In addition, combined with the actual process, the power consumption of the architecture can be analyzed. In the future, a more accurate RRAM model and analog neuron model can be introduced into FangTianSim to analyze the impact of more uncertainty of RRAM and analog circuit on SNN.

## Data Availability Statement

The original contributions presented in the study are included in the article/Supplementary Material, further inquiries can be directed to the corresponding author/s.

## Author Contributions

JW, ZW, and TS conceived the idea for the study and wrote the manuscript. JW, ZW, YL, HJ, JL, and JA developed the software. YQL and LG implemented the algorithms. JW, ZW, XZ, TS, and QL discussed about the results and analysis. All authors contributed to the article and approved the submitted version.

## Conflict of Interest

The authors declare that the research was conducted in the absence of any commercial or financial relationships that could be construed as a potential conflict of interest.

## Publisher’s Note

All claims expressed in this article are solely those of the authors and do not necessarily represent those of their affiliated organizations, or those of the publisher, the editors and the reviewers. Any product that may be evaluated in this article, or claim that may be made by its manufacturer, is not guaranteed or endorsed by the publisher.
