# Supplementary material for: FangTianSim: High-Level Cycle-Accurate Resistive Random-Access Memory-Based Multi-Core Spiking Neural Network Processor Simulator
Source: Front Neurosci. 2022 Jan 20;15:806325. doi: 10.3389/fnins.2021.806325 (PMC8811373; doi:10.3389/fnins.2021.806325)
Supplement: Supplementary file 1 [file Data_Sheet_1.docx]

Supplementary Material

# 1. Mapping method of two-layer FC and RNN

## Two-layer FCNN

As shown in **SupplementaryFigure 1**, it is a mapping method of a two-layer FCNN. The first layer is the input layer with 4 inputs, the middle layer has 3 neurons and the output layer has 2 neurons. As shown in the figure, the yellow diamond indicates the memristors used. The weight of W0 is mapped to the conductance of these memristors. The gray diamond in the figure indicates that it cannot be used by other neurons and needs to be configured as 0 because spikecomes from I0-I3 will also affect these synapses. If it is not configured as 0, synapses come from I0-I3 cause changes in the membrane potential of white neurons.3 neurons in core0 (M0-M2 axons) store the location of core1 and the label of core1 dendrites. The pulses generated by the three neurons in core0 are transmitted to core1 through the router. The red diamond in the figure indicates that the memristor is used, and the weight of W1 is mapped to the conductance of these memristors. When the number of neurons and synapses in a core can accommodate two-layer networks, the total input is less than or equal to the number of dendrites 256, and the total number of neurons (excluding the input layer) is less than or equal to the number of neurons in the core 32, a multi-layer network can be placed in a core.

## 1.2RNN

As shown in **Supplementary Figure 2**, it is anRNN, in which the input layer has four inputs, and the cyclic layer has three neurons, and each neuron has its own connection to itself. The yellow diamond is similar to the synapse of the full connection layer, corresponding to the weight W0. The cyclic layer is represented by the red figure in the figure. Each cyclic connection refers to occupying one synapse in a row of dendrites and causing the waste of other synapses in the current row; Note that the implementation of RNN must have enough free dendrites in the core where the current layer is located, and the synapses of circular layer cannot be mapped to other cores.

# 2.Neuron circuit

This section will introduce a neuron supporting positive and negative weight and negative membrane potential, which also adopts LIF model and phenomenological design method, in which the threshold conversion circuit is realized by a five-tube comparator, and the circuit realization is shown in **Supplementary Figure 4**. This neuron circuit is described in detail below.

## 2.1Phase of neuron circuit operation

The neuron circuit introduced in this section has three working phases, reset, integrate and wait, as shown in **Supplementary Figure 5(A)**. It's in the reset phase until the neuron starts working, the reset voltage of the neuron is controlled by the external circuit V_cm_, which can provide a voltage greater than 0V, as shown in **Supplementary Figure 5(B)**, the rest voltage is 0.9V; The membrane potential of neuron increases or decreases with the input pulse until it reaches a threshold voltage and generates spike, then enters wait phase to wait for the array AER circuit to receive the generated pulse; when digital AER circuit received the pulse, the neuron enters reset phase to begin the next cycle.

**2.2 2T1R cell**

As shown in **Supplementary Figure 4**, the input of the neuron has two ports connected to two 2T1R RRAM cells(see **Supplementary Figure 6** for details). 2T1R RRAM that consists of Rp, RM1 and RM2 represents the positive weight;2T1R RRAM which consists of Rd, RM3 and RM4 represents the negative weight. The 2T1R RRAM does not directly use the conductance of the memristor as the weight, but uses the memristor Rp and MOS tube RM2 to form a voltage divider circuit. The voltage divider of RM2 is used to bias the gate of the output tube RM1, and RM1 is used for output(Thick gate MOS tube of SMIC180 is used in RM2 and thin gate MOS tube of SMIC180 is used in RM1). The reason why 2T1R is used in this design is that 2T1R can be driven by a MOS tube with a smaller width-to-length ratio (the ratio of MOS channel width to channel length W/L) as the output tube, so that the output current of 2T1R is smaller, and the voltage bias of the output tube below the threshold voltage can further reduce the current and the load of neurons. This reduces the area of the neuron (using smaller CMOS tubes and integrating capacitors) and power consumption (reducing the bias current and thus the static and transient current). In this design, the output tube uses 2/1 width-length ratio.

## 3.Supplementary Figures


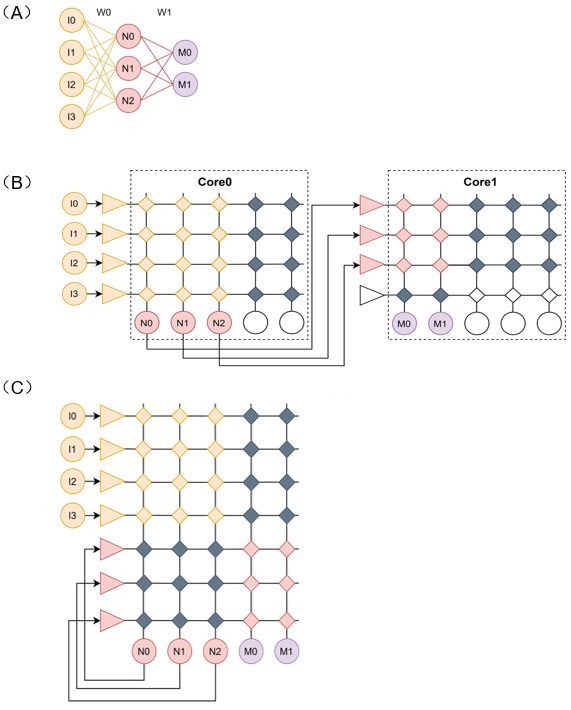


**SupplementaryFigure 1.(A)**FCNN. This network consists of input layer with 4 inputs, middle layer with 3 neurons and the output layer with 2 neurons.**(B)** FCNNmappingin two cores.In this way,the pulses generated by the three neurons in core0 are transmitted to core1 through the router**(C)** FCNNmapping in one core.If the area of the core is large enough, two-layer network can be mapped onto one core.


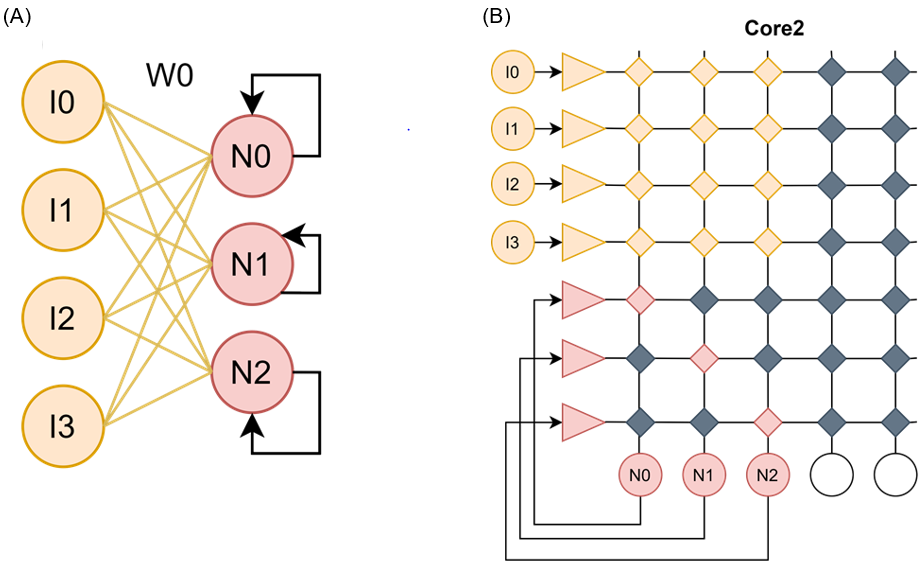


**Supplementary Figure 2.(A)** RNN.Two-layer network which has 4 inputs and 3 neurons **(B)**RNN mapping.If the area of the core is large enough, two-layer network also can be mapped onto one core.


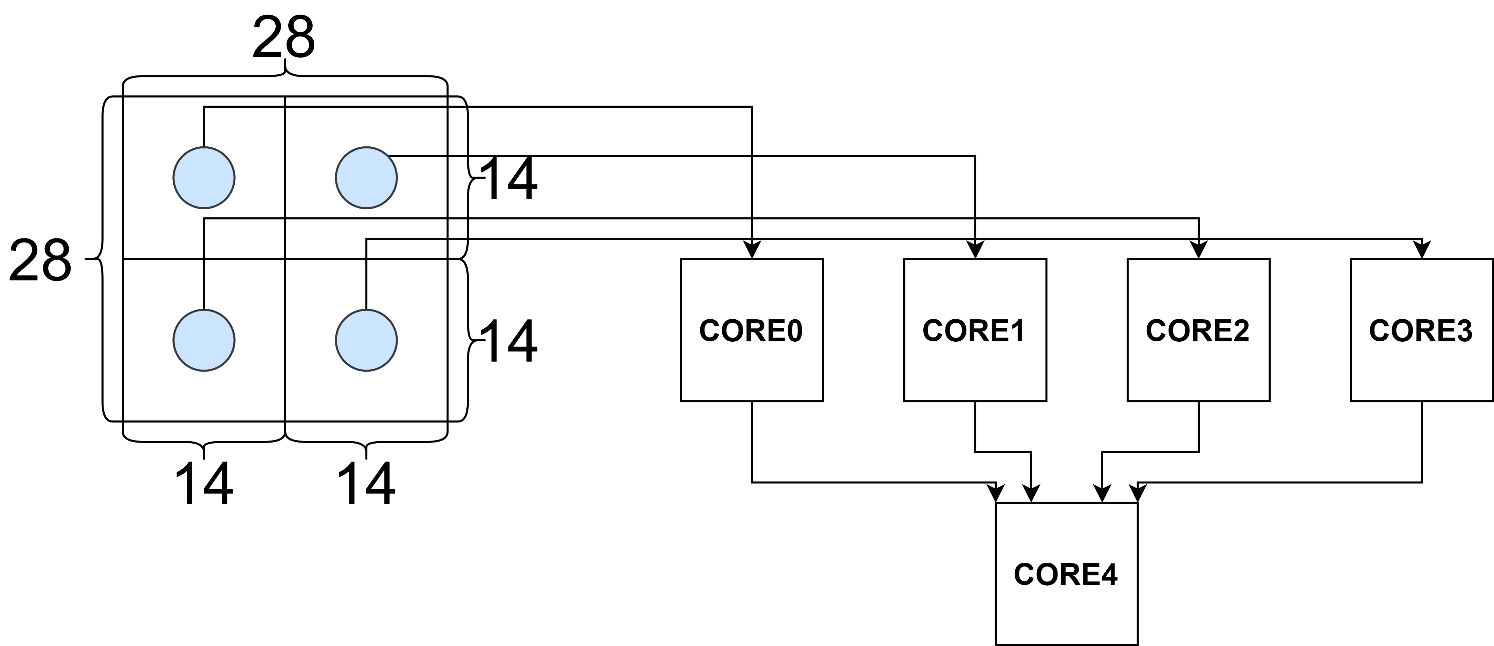


**Supplementary Figure3.**Two-layer FCNN for MNIST. The input layer of the first layer is divided into four blocks, which are input into four different cores and form an intermediate layer.


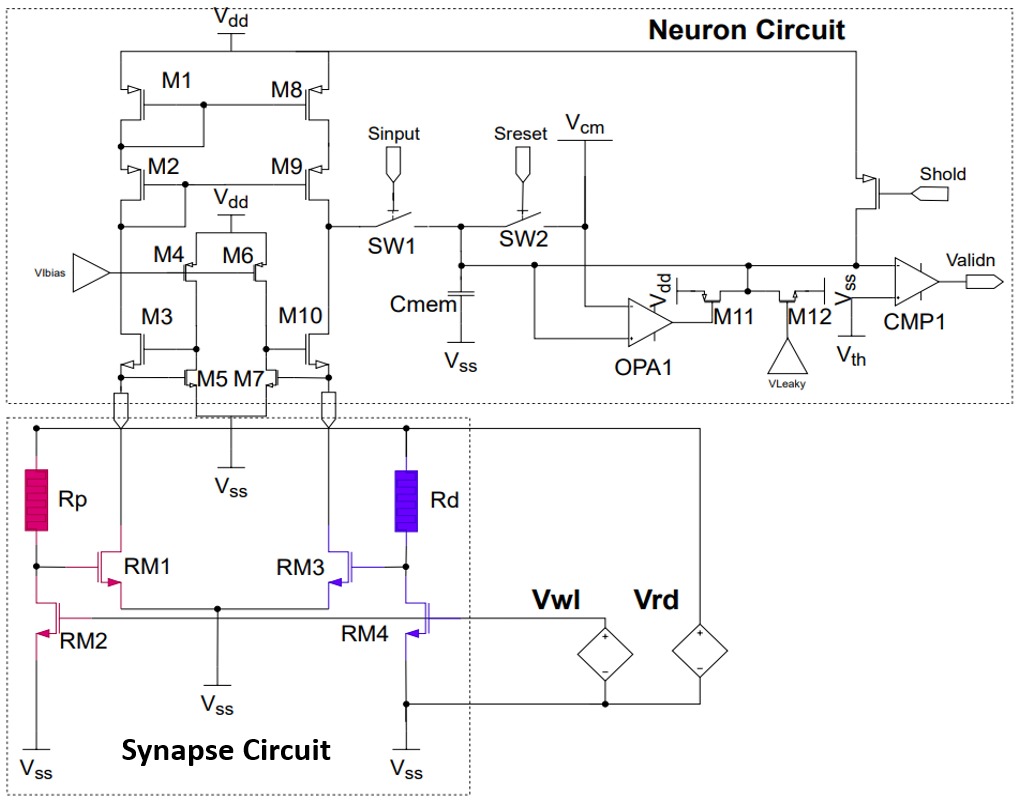


**Supplementary Figure 4.**Analog neuron circuit and synapse circuit. Red represents positive weights and blue represents negative weights.


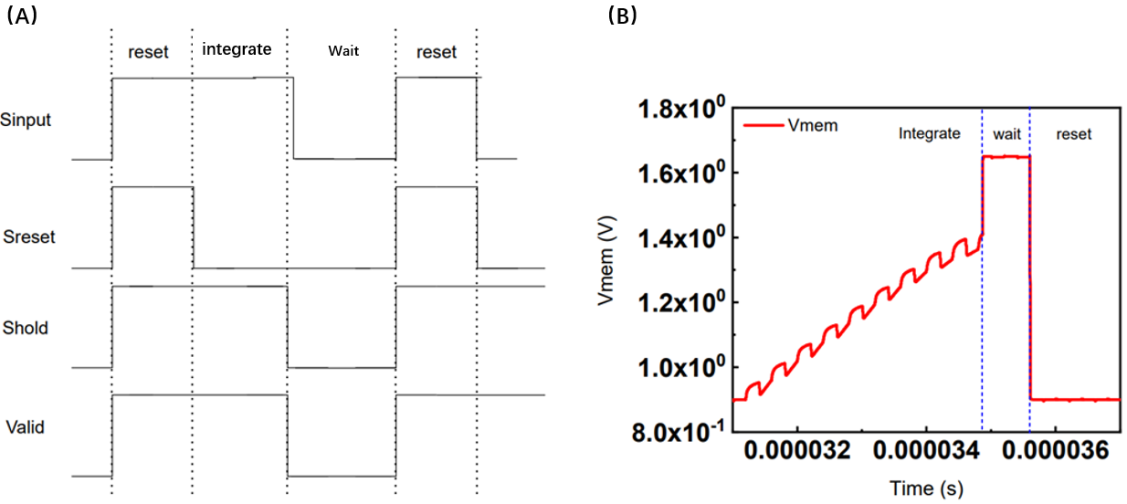


**Supplementary Figure 5.(A)**Neuron working phase. There are phases reset, integrate, and wait, which circulate with the cycle. **(B)**Changes of neuron membrane potential at different phases.


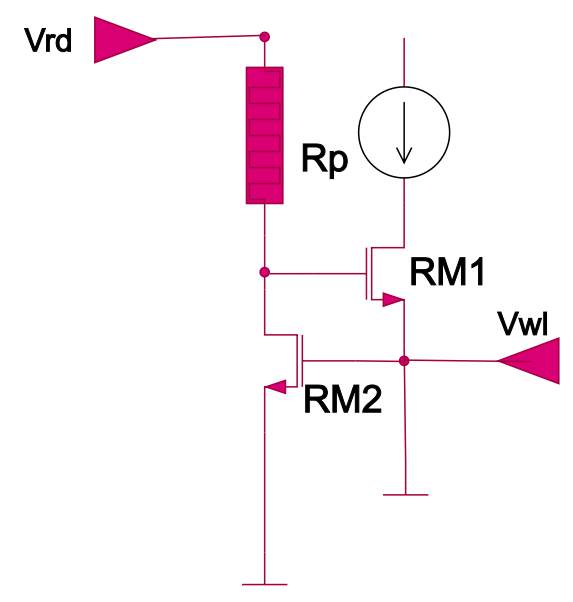


**Supplementary Figure 6.** Details of2T1R RRAM cell.
